# ERAP1 Controls the Interaction of the Inhibitory Receptor KIR3DL1 With HLA-B51:01 by Affecting Natural Killer Cell Function

**DOI:** 10.3389/fimmu.2021.778103

**Published:** 2021-11-30

**Authors:** Silvia D’Amico, Valerio D’Alicandro, Mirco Compagnone, Patrizia Tempora, Giusy Guida, Paolo Romania, Valeria Lucarini, Ombretta Melaiu, Michela Falco, Mattia Algeri, Daniela Pende, Loredana Cifaldi, Doriana Fruci

**Affiliations:** ^1^ Department of Paediatric Haematology/Oncology and of Cell and Gene Therapy, Bambino Gesù Children Hospital, Istituto di Ricovero e Cura a Carattere Scientifico (IRCCS), Rome, Italy; ^2^ Laboratory of Clinical and Experimental Immunology, Integrated Department of Services and Laboratories, Istituto di Ricovero e Cura a Carattere Scientifico (IRCCS) Istituto Giannina Gaslini, Genoa, Italy; ^3^ Laboratory of Immunology, Istituto di Ricovero e Cura a Carattere Scientifico (IRCCS) Ospedale Policlinico San Martino, Genoa, Italy; ^4^ Academic Department of Pediatrics (DPUO), Bambino Gesù Children Hospital, Istituto di Ricovero e Cura a Carattere Scientifico (IRCCS), Rome, Italy; ^5^ Department of Clinical Sciences and Translational Medicine, University of Rome “Tor Vergata”, Rome, Italy

**Keywords:** ERAP1, NK cells, NK cell immunotherapy, HLA class I, KIR, cancer

## Abstract

The endoplasmic reticulum aminopeptidase ERAP1 regulates innate and adaptive immune responses by trimming peptides for presentation by major histocompatibility complex (MHC) class I molecules. Previously, we have shown that genetic or pharmacological inhibition of ERAP1 on murine and human tumor cell lines perturbs the engagement of NK cell inhibitory receptors Ly49C/I and Killer-cell Immunoglobulin-like receptors (KIRs), respectively, by their specific ligands (MHC class I molecules), thus leading to NK cell killing. However, the effect of ERAP1 inhibition in tumor cells was highly variable, suggesting that its efficacy may depend on several factors, including MHC class I typing. To identify MHC class I alleles and KIRs that are more sensitive to ERAP1 depletion, we stably silenced ERAP1 expression in human HLA class I-negative B lymphoblastoid cell line 721.221 (referred to as 221) transfected with a panel of KIR ligands (i.e. HLA-B*51:01, -Cw3, -Cw4 and -Cw7), or HLA-A2 which does not bind any KIR, and tested their ability to induce NK cell degranulation and cytotoxicity. No change in HLA class I surface expression was detected in all 221 transfectant cells after ERAP1 depletion. In contrast, CD107a expression levels were significantly increased on NK cells stimulated with 221-B*51:01 cells lacking ERAP1, particularly in the KIR3DL1-positive NK cell subset. Consistently, genetic or pharmacological inhibition of ERAP1 impaired the recognition of HLA-B*51:01 by the YTS NK cell overexpressing KIR3DL1*001, suggesting that ERAP1 inhibition renders HLA-B*51:01 molecules less eligible for binding to KIR3DL1. Overall, these results identify HLA-B*51:01/KIR3DL1 as one of the most susceptible combinations for ERAP1 inhibition, suggesting that individuals carrying HLA-B*51:01-like antigens may be candidates for immunotherapy based on pharmacological inhibition of ERAP1.

## Introduction

Natural killer (NK) cells provide the first important line of defence against infections and malignancies through direct recognition and killing of altered cells (1, 2). The effector function of NK cells is tightly regulated through the balance of activating and inhibitory signals resulting from interaction with surrounding cells (1–3). Accordingly, NK cells express activating and inhibitory receptors that bind to major histocompatibility complex (MHC) class I molecules, including CD94/NKG2 receptors and killer cell immunoglobulin-like receptors (KIRs) in humans, and Ly49 in mice (4). The engagement of inhibitory receptors by MHC class I molecules on healthy cells normally suppresses the activation of autologous NK cells (4). However, if these interactions are disrupted, as a result of downregulation of MHC class I or presentation of an antagonist peptide, inhibition is lost resulting in NK-cell activation and lysis of target cells (5). Inhibitory receptors typically recognise a group of classical and non-classical MHC class I molecules with specific amino acid sequences at positions 77-83 of the alpha-1 domain (6). Based on these sequences, some HLA-B and HLA-A molecules are classified as Bw4 allotypes (6). In general, KIR2DL1 binds HLA-C alleles with lysine at position 80; KIR2DL2 and KIR2DL3 bind HLA-C alleles with asparagine at position 80; KIR3DL1 recognizes HLA-B and HLA-A alleles expressing Bw4 epitope; KIR3DL2 recognizes HLA-A*03 and HLA-A*11; CD94-NKG2A receptor interacts with non-classical HLA-E molecule (7). Inhibition through CD94-NKG2A requires stabilization of the HLA-E heavy chain through association with β2m and short peptide ligands that result from cleavage of the signal sequences of “permissive” HLA class I alleles (i.e., classical HLA-A, -B, -C, and nonclassical HLA-G) (8, 9). In the absence of the “permissive” HLA class I alleles, HLA-E molecules are not functionally expressed on the cell surface. This is consistent with the observation that HLA class I-negative human B lymphoblastoid cell line 721.221 (hereafter referred to as 221) expresses HLA-E primarily intracellularly and only marginally on the cell surface (10). Indeed, transfection of 221 cells with signal peptides derived from “permissive” HLA class I molecules has been shown to induce surface expression of HLA-E (11).

KIRs also exhibit selectivity for peptides bound to MHC class I molecules. Indeed, crystal structures of KIR-HLA class I complexes show that KIRs directly contact residues 7 and 8 of peptides bound to MHC class I molecules (6). Interestingly, changes in these positions can promote or abrogate KIR binding and, ultimately, NK cell reactivity to target cells (12–14). This was originally demonstrated for KIR3DL1 and HLA-B*27:05, and then observed for other inhibitory KIRs, including KIR2DL1, KIR2DL2, KIR2DL3 and KIR3DL2 (12, 15–19). Similar to KIRs, CD94/NKG2A inhibitory signalling is also dependent on the peptide presented by HLA-E molecules (11, 20–22), suggesting that both inhibitory KIRs and NKG2A are peptide selective.

ERAP1 is a multifunctional endoplasmic reticulum (ER) aminopeptidase that shape the peptide repertoire presented by classical and non-classical MHC class I molecules (23). ERAP1 gene is highly polymorphic with multiple combinations of SNPs, called haplotypes, affecting functional enzymatic activity (24–26). Indeed, genome wide association studies (GWAS) have revealed 10 different haplotypes (Hap1 to Hap10) with a frequency greater than 1%, and several functional SNPs associated with susceptibility to autoimmune diseases in individuals carrying the HLA class I-risk alleles, such as HLA-B27 for ankylosing spondylitis, HLA-Cw6 for psoriasis, and HLA-B51 for Behçet’s disease (23).

Previously, we have demonstrated that genetic and pharmacological inhibition of ERAP1 on murine and human tumor cell lines perturbs their ability to engage several classes of inhibitory receptors by their ligands, including Ly49C/I and killer cell Ig-like receptors (KIR), respectively, leading to NK cell killing (27, 28). Notably, in both cases, replacement of endogenous peptides with high-affinity peptides was sufficient to restore an NK-protective effect of MHC class I *via* the inhibitory NK cell receptors KIRs and CD94-NKG2A (27, 28). In addition, we also found that ERAP1 inhibition enhanced the ability of NK cells to kill newly established human lymphoblastoid cell lines from autologous or allogeneic sources (28), thus promoting NK cell-mediated cytotoxic activity against target cells that would not be expected due to KIR-KIR ligand matching. Of note, the use of donor-derived alloreactive NK cells has been shown to be particularly effective for leukaemia patients undergoing haploidentical hematopoietic stem cell transplantation (HSCT) to eradicate malignant cells (29).

However, the effect of ERAP1 inhibition in tumor cells and LCLs was highly variable, suggesting that it may depend on MHC class I typing and/or *ERAP1* genotype (26). To identify KIR-HLA class I interactions more sensitive to ERAP1 inhibition, we stably reduced ERAP1 expression in HLA class I-negative 221 cells transfected with a panel of KIR ligands (i.e. HLA-B*51:01, -Cw3, -Cw4 and -Cw7), or HLA-A2 which does not bind any KIR, and tested their ability to induce NK cell degranulation and cytotoxicity. We show that genetic and pharmacological inhibition of ERAP1 renders 221-B*51:01 cells susceptible to killing by NK cells, due to impairment of KIR3DL1/HLA-B51 interaction. In the clinical setting, our data suggest ERAP1 inhibition as a novel NK cell-based immunotherapy strategy for patients with functional ERAP1 and favourable HLA class I typing.

## Material and Methods

### Cell Lines

YTS and 221 parental and transfectant cell lines were kindly provided by Peter Doherty, Jose A. Lopez de Castro and Patrizio Giacomini. Transfectant cell lines were maintained in complete medium with the addition of 0.75 mg/mL Geneticin (G418, Gibco by ThermoFisher) for 221-A2, 221-B*51:01, 221-Cw3 and 221-Cw4, 300 µg/mL Hygromycin B (Sigma Aldrich) for 221-Cw7, and 10 µg/mL Blasticidin (Sigma Aldrich) for YTS-KIR3DL1*001. Cells infected with pLKO.1 plasmids were selected with 3 µg/mL Puromycin. When indicated, 221 transfectants were treated with 30 µM LeuSH (Sigma Aldrich) for 24 hours.

### ERAP1 Haplotype

RNA isolated from 1×10^6^ 221 cells with TRIzol Reagent (by ThermoFisher) was used to generate cDNA with the SuperScript IV Reverse Transcriptase (Invitrogen, by ThermoFisher). ERAP1 was amplified from cDNA, using Expand™ High FidelityPLUS PCR System (Roche, Sigma Aldrich) and the following primers were used (Sigma Aldrich): Fw 5′-ATGGTGTTTCTGCCCCTCAAATGGT-3′; Rev 5′-TTACATACGTTCAAGCTTTTCAC-3′. The PCR amplicon was cloned by TA cloning method with pGEM-T Easy Vector System (Promega) and sequenced with 4 different sequencing primers (**Table S2**) to identify individual haplotypes.

### Western Blotting

Equal amounts of protein extracts were resolved on 8% polyacrylamide gel and transferred on nitrocellulose membranes (Amersham Systems, Ge Healthcare Life Sciences). Filters were blocked with 5% (v/v) non-fat dry milk for 1 hour at room temperature, and then blotted with rabbit R5996-4 antibody to recognize MHC class I (kindly provided by Prof. Tanigaki), and murine 6H9 and 3F5 antibodies to recognize ERAP1 and ERAP2, respectively (generously provided by Prof van Endert). Anti-ERp57 was used as loading control. After extensive washing with TBST, filters were incubated with peroxidase-coupled secondary antibody for 1 hour at room temperature. Reactivity was detected with the ECL Western Blotting Detection Kit (Amersham Systems, Ge Healthcare Life Sciences) and the protein bands were quantified using Image J.

### Flow Cytometry

Surface expression of MHC class I molecules was determined by staining cells with the W6/32 mouse primary antibody (kindly provided by Dr Giacomini), which recognize classical HLA allotypes (-A, -B, -C) and the non-classical HLA-E. W6/32 positive cells were detected with secondary antibody Goat F(ab’)2 Fragment Anti-Mouse IgG (Fcγ)-FITC (Beckman Coulter). For allele-specific HLA class I staining, cells were stained with three allele-specific HLA class I antibodies: mouse anti-HLA-A2 (clone BB7.2) for 221-A2 cells, FITC-conjugated IgG2a anti-Bw4 for 221-B*51:01 cells and FITC-conjugated IgG3 anti-Bw6 for 221-Cw3 and 221-Cw7 cells.

In degranulation assays, NK cells were stained with CD56-PE-Cy7 clone B159 and CD107a-BV421 clone H4A3 (BD Bioscience), KIR2DL1/S1-PE-Cy5.5 clone EB6B, KIR2DL2/L3/S2-PE clone GL183 (Beckman Coulter), KIR3DL1-APC clone DX9 (R&D Systems). NKG2A expression was detected with anti-NKG2A-FITC clone REA110 (Miltenyi Biotec) or anti-NKG2A-Alexa Fluor700 conjugated clone a131411 (R&D Systems). Samples were acquired using FACSCanto II or LSRFortessa X-20 (BD Bioscience) and the resulting data were subsequently analysed using FlowJo V 10.2 software.

### Lentivirus Production and Cell Infection

Lentiviral particles were generated in HEK293T cells transfected with packaging plasmid pCMV-dR8.74, envelope plasmid VSV-G/pMD2.G, and pLKO.1 plasmid containing a non-target shRNA control sequence (Sigma-Aldrich SHC002) or the ERAP1 shRNA (clone ID: TRCN0000060542) targeting human ERAP1 (Sigma-Aldrich) by using the TransIT-293 Transfection Reagent (Mirus Bio). Viral supernatant was collected 72 hours post-transfection, filtered and used for 221 infection after addition of 8 µg/mL Hexadimethrine bromide (Sigma Aldrich). Cells were infected by spin inoculation method. Briefly, cells were centrifuged in lentiviral medium at 1800 rpm at 32°C for 45 minutes.

### NK Cell Isolation

Human NK cells were isolated from peripheral blood mononuclear cells (PBMC) of healthy donors co-expressing KIR2DL1, KIR2DL2 and KIR3DL1 by using the RosetteSep NK cell enrichment mixture method (Stem-Cell Technologies). Blood from buffy coat bags was incubated with the NK cell enrichment mixture (50 µL per mL of blood) for 20 minutes. Enriched NK cells were then isolated by centrifugation in the Ficoll separation medium (Amersham). CD56^+^ NK cells obtained a purity greater than 90% were plated at a concentration of 1x10^6^ cells/mL in complete RPMI-1640 medium with the addition of 200 IU/mL of recombinant human IL-2 (PeproTech) over night at 37°C and then used for functional experiments.

### Degranulation and Cytotoxicity Assays

Degranulation assay was performed by co-culturing target and NK cells at a 1:1 ratio for 3 hours at 37°C in the presence of anti-CD107a; GolgiStop solution (BD Bioscience) was added after the first hour of co-culture. Degranulation levels were evaluated also in NK cell subsets single positive (sp) for KIR expression and negative for NKG2A expression.

The cytotoxic activity of YTS and YTS overexpressing KIR3DL1 cells was evaluated using the CytoTox 96 Non-Radioactive Cytotoxicity Assay kit (Promega), a colorimetric assay that measures the concentration of lactate dehydrogenase (LDH), a stable cytosolic enzyme released upon cell lysis and considered an indicator of cytotoxicity. Accordingly to the protocol, target cells (221-B*51:01-shCTRL or 221-B*51:01-shERAP1) were co-cultured with effectors (YTS or YTS-KIR3DL1) at different effector to target (E:T) cell ratios in a 96-well U-bottom plate and incubated at 37°C for 4 hours. Subsequently, 50 *μ*l per well of supernatants was collected for detecting LDH release in the Microplate Imaging System at an absorbance of 490 nm. As controls, spontaneous LDH release was evaluated by incubation of NK cells or target cells alone, and maximum LDH release was assessed by incubation of target cells in 0.1% Triton X-100. The percentage of specific LDH release was calculated as follows: experimental optical density (OD) - effector spontaneous OD - target spontaneous OD)/(target maximum OD - target spontaneous OD).

### Statistical Analysis

Statistical significance was assessed by two-tailed *t*-test or multiple *t*-test using PRISM GraphPad software. P values not exceeding 0.05 were considered to be statistically significant.

## Results

### 721.221 Transfectant Cells: A Suitable Model to Investigate the Role of ERAP1 in KIR-HLA Class I Interaction

**Figure 1:**
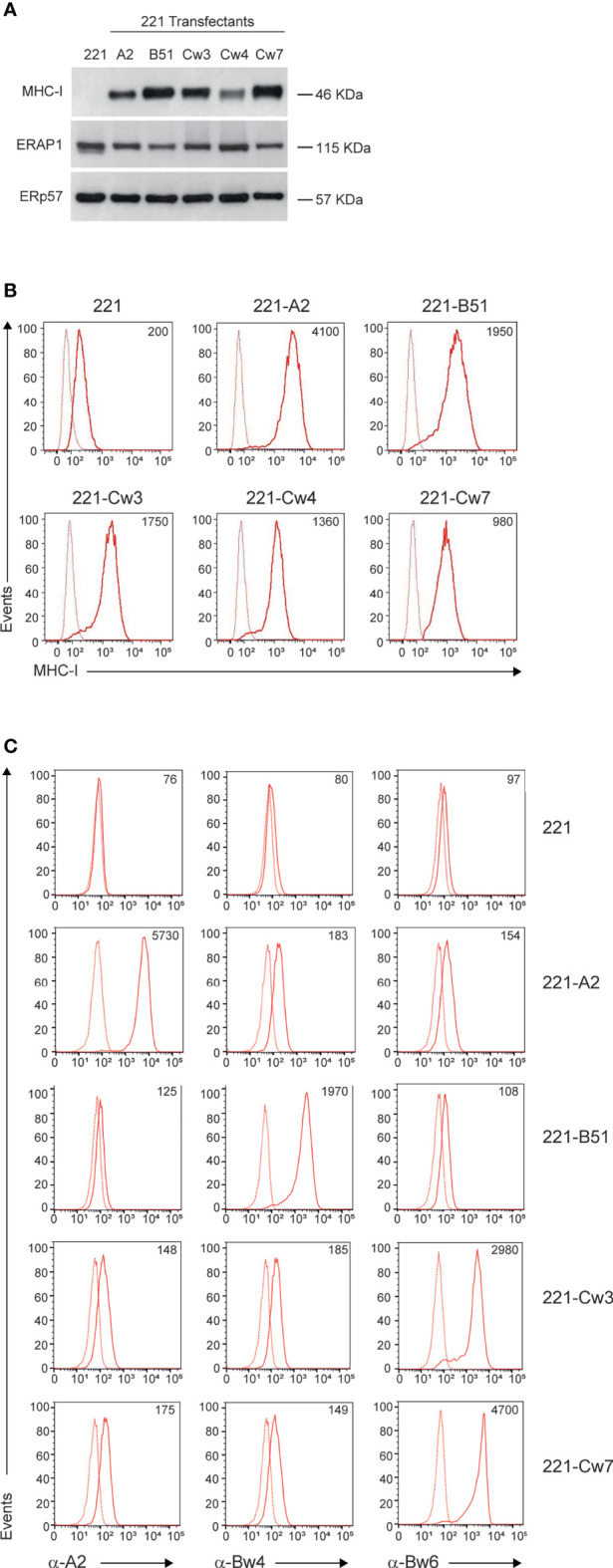
MHC class I and ERAP1 expression in 221 transfectant cells. **(A)**, Representative immunoblotting analysis of MHC class I and ERAP1 expression in 221 parental and transfectant cells. An ERp57 antibody was used for normalization. **(B, C)**, Representative flow cytometric analyses of HLA class I cell surface expression in the indicated cell lines with the pan-anti HLA class I W6/32 antibody **(B)** and anti-HLA-A2 (BB7.2), anti-HLA-Bw4 and anti-HLA-Bw6 **(C)** antibodies. In **(B, C)**, isotype-matched negative control antibodies are displayed as dotted lines. MFI value for specific antibody staining are included.

To identify KIR/HLA class I combinations susceptible to ERAP1 inhibition, we took advantage of 221 cell line, which does not express the endogenous HLA class I alleles (HLA-A, -B and -C), and 221 transfectant cells overexpressing ligands of inhibitory KIRs: HLA-B*51:01 for KIR3DL1, HLA-Cw4 for KIR2DL1, and HLA-Cw3 and HLA-Cw7 for KIR2DL2 and KIR2DL3. As control we used 221 transfectant cells overexpressing HLA-A2, which does not bind any KIR. MHC class I expression was evaluated in 221 parental and transfectant cells by western blotting and flow cytometric analyses (**Figure 1**). As expected, no MHC class I expression was detected in 221 cells, whereas all 221 transfectants expressed comparable levels of MHC class I molecules (**Figure 1A**). Accordingly, flow cytometric analysis revealed high levels of cell surface MHC class I expression in all 221 transfectant cells as assessed by the use of the pan-anti HLA class I W6/32 antibody (**Figure 1B**). Conversely, the parental 221 cells showed low, but not negative MHC class I expression, due to the detection of endogenous HLA-E molecules by W6/32 antibody (**Figure 1B**) (30). To further characterize the expression of individual HLA class I alleles, 221 transfectant cells were stained with anti-HLA-A2, anti-Bw4 and anti-Bw6 monoclonal antibodies (**Figure 1C**). Specifically, anti-HLA-A2 (BB7.2) antibody recognizes the human HLA-A2 antigen, whereas anti-Bw4 and anti-Bw6 antibodies bind to the region encompassing residues 77-83 of the alpha-1 helix of HLA class I molecules, thereby discriminating between the two sets of HLA-B allotypes (31). The anti-Bw6 antibody, which also recognizes C1 allotypes, was used to characterize 221-Cw3 and 221-Cw7 transfectant cells for which no allele-specific antibody is available (32). As expected, no reactivity was detected in 221 parental cells, whereas specific reactivity was observed in the distinct transfectants: BB7.2 antibody strongly bound 221-A2 transfected cells, Bw4-reactive antibody bound 221-B*51:01 transfectant cells, and Bw6-reactive antibody bound 221-Cw3 and 221-Cw7 (**Figure 1C**). No or a weak specific binding was observed between antibodies in transfectants expressing unrelated antigens (**Figure 1C**). Unfortunately, no allele specific antibody is available to further characterize 221-Cw4 cells.

Haplotype analysis of ERAP1 revealed that 221 cells harbor the Hap1/Hap8 combination (**Table S1**), namely coding for a high (Hap1) and an intermediate (Hap8) aminopeptidase activity (33). Consistently, ERAP1 protein was expressed at high levels in all 221 transfectants (**Figure 1A**).

### Genetic Inhibition of ERAP1 Does Not Affect Surface Expression of HLA Class I Molecules in 221 Transfectant Cells

**Figure 2:**
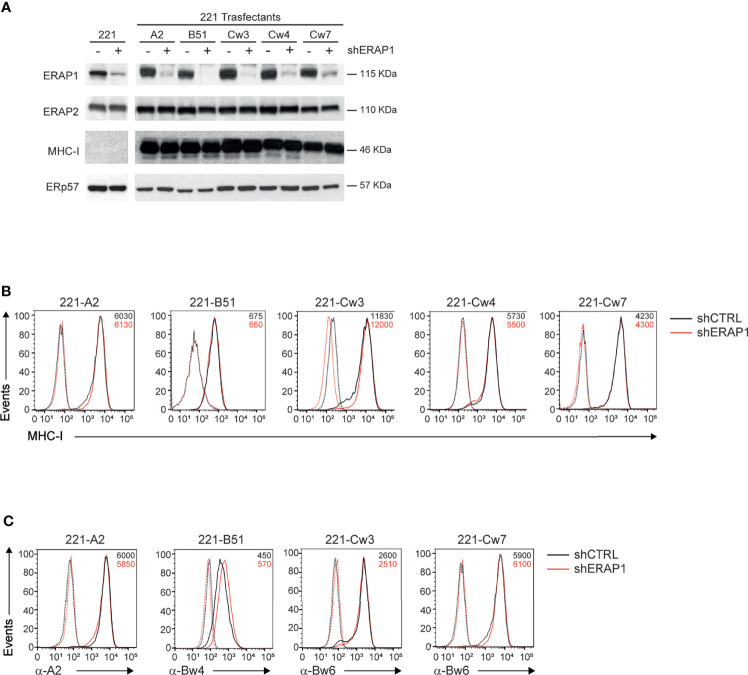
Inhibition of ERAP1 does not affect HLA class I surface expression in 221 transfectant cells. **(A)**, Representative immunoblotting analysis of ERAP1, ERAP2 and HLA class I expression in the indicated 221 parental and transfectant cells transduced with lentiviral vectors encoding either control shRNA (-) or ERAP1 shRNA (+). An ERp57 antibody was used for normalization. **(B, C)**, Representative flow cytometric analyses of HLA class I expression in the indicated cells with the pan-anti HLA class I W6/32 antibody **(B)** and anti-HLA-A2 (BB7.2), anti-HLA-Bw4, and anti-HLA-Bw6 antibodies **(C)**. Isotype-matched negative control antibodies are as dotted lines. MFI value for specific antibody staining are indicated.

ERAP1 expression was interfered in 221 parental and transfectant cells by infection with lentiviral particles encoding small hairpin RNA targeting human ERAP1 (shERAP1) or a control sequence (shCTRL). Compared to shCTRL-transduced cells, ERAP1 protein expression was strongly reduced in all shERAP1-transduced 221 cells overexpressing HLA class I antigens (**Figure 2A**). Conversely, no change in ERAP2 protein expression was detected in shERAP1 cells, indicating the high specificity of the shERAP1 lentiviral construct (**Figure 2A**). No change in total and surface MHC class I expression was detected in ERAP1-depleted cells compared to controls (**Figures 2A, B**). Similar results were obtained with allele-specific antibodies (**Figure 2C**).

### Genetic Inhibition of ERAP1 Enhances NK Cell-Mediated Killing of 221-B*51:01 Transfectant Cells

**Figure 3:**
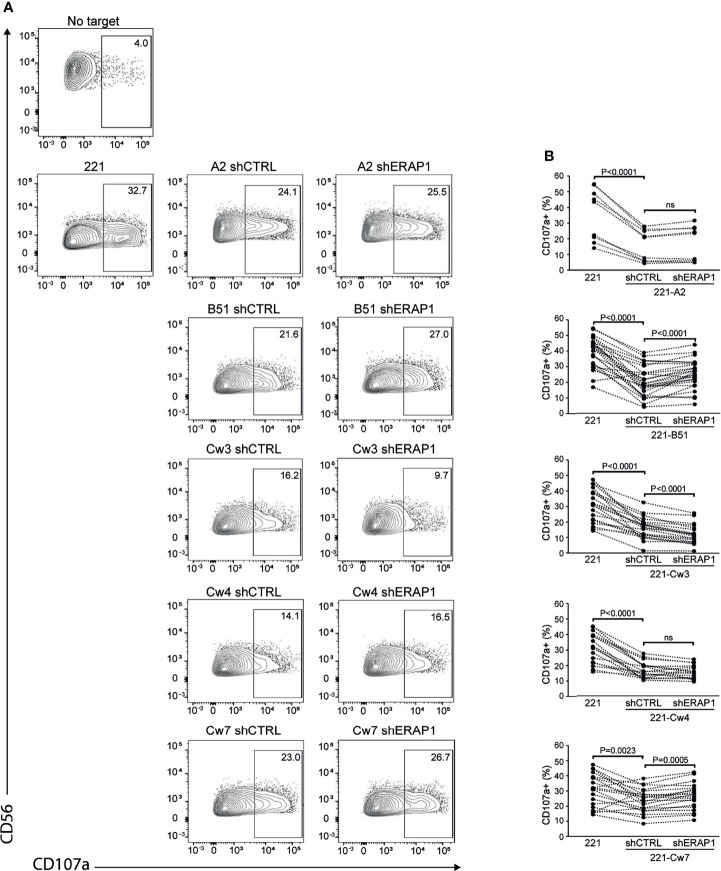
Inhibition of ERAP1 enhances NK cell killing of 221-B*51:01 cells. **(A)**, Example of degranulation by human CD56^+^ NK cells from one healthy donor expressing KIR2DL1, KIR2DL3, and KIR3DL1 measured as CD107a cell surface expression in response to the indicated cells. The percentage of CD107a^+^ cells within the indicated NK cell subsets is shown. **(B)** Summary of NK cell degranulation from donors after stimulation with 221 parental and transfectant cells in response to ERAP1 inhibition. *P* values, calculated by two-tailed paired Student’s *t*-test. ns, not significant.

Next, 221-shERAP1 transfectant cells were assayed for the ability to induce NK cell degranulation. NK cells freshly isolated from healthy donors were co-cultured with shERAP1 cells in a 1:1 effector:target ratio, and CD107a expression on NK cells was determined by flow cytometric analysis. A different number of KIR2DL1-, KIR2DL3- and KIR3DL1-expressing donors was tested with various transfectant cells. As expected, CD107a expression was increased on NK cells after stimulation with the HLA class I-negative target 221 cell line, and significantly reduced by stimulation with shCTRL-transduced 221 transfectant cells (**Figures 3A, B**). The mean frequency of CD107a^+^ cells in different healthy donors was 16.1 ± 10 *vs* 35.8 ± 16.8 for 221-A2-shCTRL *vs* 221, 20.5 ± 9.7 *vs* 38,9 ± 9.9 for 221-B*51:01-shCTRL *vs* 221, 16.1 ± 7.1 *vs* 30.9. ± 10.4 for 221-Cw3-shCTRL *vs* 221, 16.6 ± 5.4 *vs* 30.9 ± 9.7 for 221-Cw4-shCTRL *vs* 221, and 23.6 ± 7.8 *vs* 30.6 ± 10.8 for 221-Cw7-shCTRL *vs* 221 (**Figures 3A, B**). These data indicate that HLA class I expression conferred strong protection from NK lysis mediated by inhibitory KIRs.

Interestingly, inhibition of ERAP1 significantly increased NK cell stimulation by 221-B*51:01-shERAP1 cells compared to 221-B*51:01-shCTRL cells (mean frequency of CD107a^+^ cells was 25.0 ± 9.2 *vs* 20.5 ± 9.7 for 221-B*51:01-shERAP1 *vs* 221-B51shCTRL), and by 221-Cw7-shERAP1 compared to relative controls (26.4 ± 8.6 *vs* 23.6 ± 7.8 for 221-Cw7-shERAP1 *vs* 221-Cw7-shCTRL) (**Figures 3A, B**). No change in CD107a expression was detected after stimulation by 221-A2-shERAP1 and 221-Cw4-shERAP1 cells, whereas a weak but significant decrease was detected after stimulation by 221-Cw3-shERAP1 compared to 221-Cw3-shCTRL (**Figures 3A, B**).

### Genetic Inhibition of ERAP1 Enhances NK Cell-Mediated Killing of 221-B*51:01 by Impairing Engagement of KIR3DL1 and NKG2A

**Figure 4:**
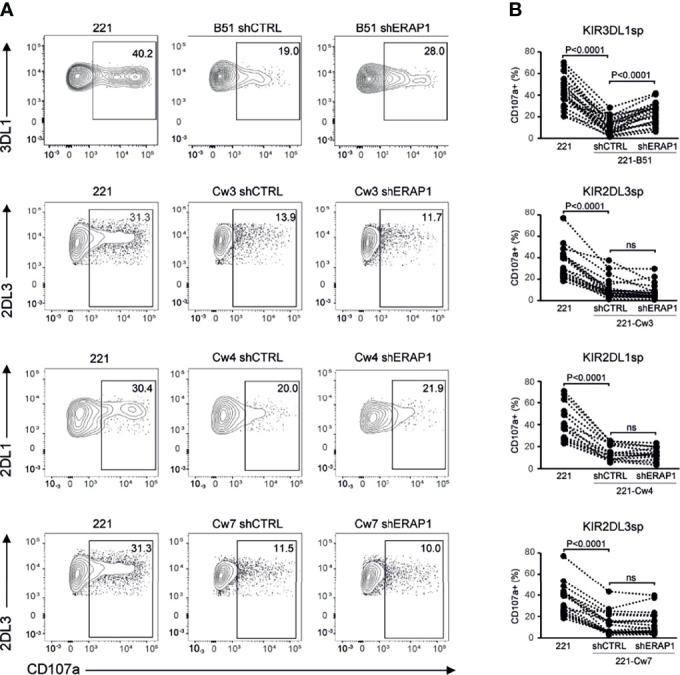
Inhibition of ERAP1 enhanced NK cell killing of 221-B*51:01 cells by affecting the engagement of KIR3DL1. **(A)**, Example of degranulation by human CD56^+^ NK cells from one healthy donor co-expressing KIR2DL1, KIR2DL3, and KIR3DL1; CD107a cell surface expression was measured in specific KIR single positive (KIRsp) subsets (KIR3DL1sp for 221-B51 cells, KIR2DL1sp for 221-Cw4 cells and KIR2DL3 for 221-Cw3 and -Cw7 cells) in response to ERAP1-inhibited 221 transfectants and control cells. The percentage of CD107a^+^ cells within the indicated NK cell subsets is shown. **(B)**, Summary of NK cell degranulation in the indicated KIRsp NK subsets from healthy donors after stimulation with 221 parental and transfectant cells in response to ERAP1 inhibition. *P* values, calculated by two-tailed paired Student’s *t*-test. ns, not significant.

**Figure 5:**
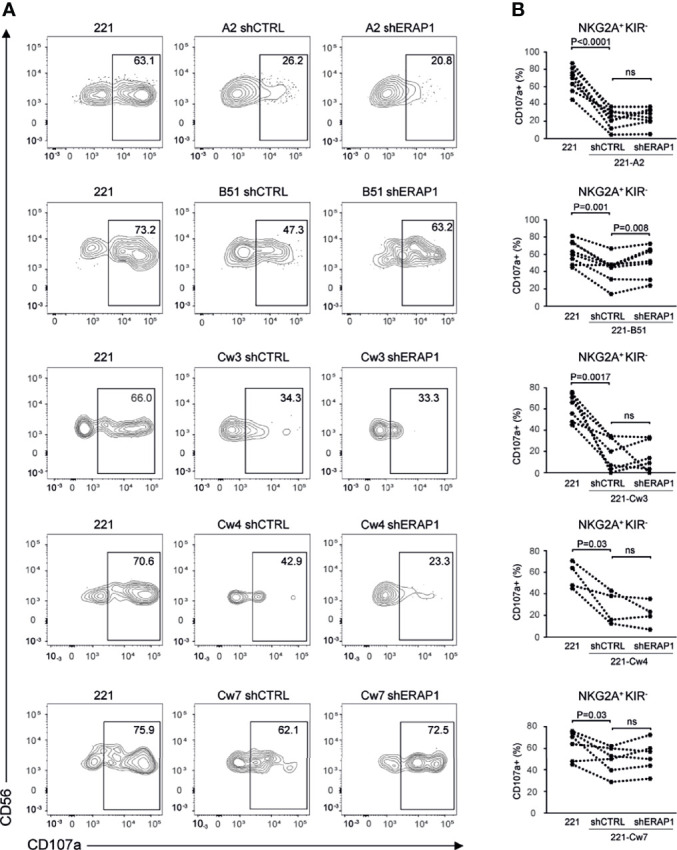
Inhibition of ERAP1 enhanced NK cell killing of 221-B*51:01 cells by affecting the engagement of NKG2A. **(A)**, Example of degranulation by human CD56^+^ NK cells from one healthy donor; CD107a cell surface expression was measured in NKG2A positive subset negative for KIR3DL1, KIR2DL1 and KIR2DL3 in response to ERAP1-inhibited 221 transfectants and control cells. The percentage of CD107a^+^ cells within the indicated NK cell subset is shown. **(B)**, Summary of NK cell degranulation in the NKG2A positive KIR negative NK subset from healthy donors after stimulation with 221 parental and transfectant cells in response to ERAP1 inhibition. *P* values, calculated by two-tailed paired Student’s *t*-test. ns, not significant.

To identify inhibitory receptors involved in NK cell activation in response to ERAP1-depleted 221 transfectant cells, CD107a expression was assessed in distinct subsets of NK cells. We focused on the three inhibitory receptors KIR2DL1, KIR2DL3 and KIR3DL1 specific for the ligands expressed on 221 transfectant cells, and CD94-NKG2A receptor. Unavailability of a specific antibody did not allow analysis of KIR3DL2. NK cells co-cultured with shERAP1 or shCTRL 221 transfectant cells were stained with a panel of specific antibodies and analyzed by flow cytometry. CD107a expression was evaluated in NK cell subsets expressing inhibitory KIRs specific for each transfectant, (i.e., single positive KIR3DL1 (KIR3DL1sp) for 221-B*51:01, KIR2DL1sp for 221-Cw4, and KIR2DL3sp for 221-Cw3 and 221-Cw7 cells), and in NK cells expressing NKG2A but negative for KIRs (NKG2A^+^KIR^-^). The gating strategy used to evaluate the contribution of single inhibitory KIRs and NKG2A^+^KIR^-^ is shown in **Figure S1**. NK cell subsets expressing single inhibitory KIRs were all responsive to stimulation with 221 cells (**Figures 4A, B**). CD107a expression of the KIRsp NK cell subsets decreased significantly (up to 3.6 fold) when stimulated with 221 transfectant cells (**Figures 4A, B**). The average percentages of CD107a^+^ KIRsp subsets co-cultured with 221 cells versus 221-B*51:01-shCTRL, 221-Cw3-shCTRL, 221-Cw4-shCTRL, or 221Cw7-shCTRL cells were 44.9 ± 16.5 *vs* 12.5 ± 12.7 for KIR3DL1sp subset, 34.6 ± 14.5 *vs* 11.3 ± 9.5 for KIR2DL3sp, 33.8 ± 14.6 *vs* 14.5 ± 8.5 for KIR2DL1sp, and 32.6 ± 14.0 *vs* 11.8 ± 9.9 for KIR2DL3sp, respectively (**Figures 4A, B**). Significant upregulation (up to 2.1 fold) of CD107a expression in KIR3DL1sp NK cell subset was detected after stimulation with 221-B*51:01-shERAP1 compared with 221-B*51:01-shCTRL, whereas no significant stimulation was observed for the others ERAP1-inhibited 221 transfectants compared with their relative controls (**Figures 4A, B**). The mean frequency of CD107a cells was 25.8 ± 14.6 *vs* 12.5 ± 12.7 for 221-B*51:01-shERAP1 vs 221-B*51:01-shCTRL in KIR3DL1sp subset; 8.1 ± 7.3 *vs* 11.3 ± 9.5 for 221-Cw3shERAP1 *vs* 221-Cw3shCTRL in KIR2DL3sp subset, 12.9 ± 5.9 *vs* 14.5 ± 8.5 for 221-Cw4-shERAP1 *vs* 221-Cw4-shCTRL in KIR2DL1sp subset and 12.1 ± 10.0 *vs* 11.8 ± 9.9 for 221-Cw7-shERAP1 *vs* 221-Cw7shCTRL in KIR2DL3sp subset (**Figure 4B**). NKG2A^+^KIR^-^ cells were also responsive to stimulation with 221 cells (**Figures 5A, B**). CD107a expression decreased significantly when stimulated with 221 transfectant cells, but with different extent (**Figures 5A, B**). Specifically, the mean frequency of CD107a in NKG2A^+^KIR^-^ cells was reduced up to 3 fold when stimulated with 221-A2-shCTRL or 221-Cw3-shCTRL compared with 221 cells (68.9 ± 13.9 *vs* 22.9 ± 10.7 for 221 *vs* 221-A2-shCTRL, and 62.1 ± 12.7 *vs* 18.8 ± 15.3 for 221 *vs* 221-Cw3-shCTRL), and up to 1.5 fold when stimulated with 221-B*51:01-shCTRL and 221-Cw4-shCTRL (64.6 ± 13.7 *vs* 40.8 ± 22.3 for 221 *vs* 221-B51-shCTRL, and 58.3 ± 11.2 *vs* 25.1 ± 14.2 for 221 *vs* 221-Cw4-shCTRL). A weak but still significant decrease was detected for 221-Cw7-shCTRL compared to 221 cells (63.1 ± 12.3 *vs* 50.5 ± 12.3 for 221 *vs* 221-Cw7-shCTRL) (**Figures 5A, B**). These differences reflect the degree of affinity of peptide ligands of HLA-E molecules resulting from the cleavage of the signal sequences of “permissive” and “non-permissive” HLA class I alleles (8). Similar to what observed for KIR3DL1sp, a significant upregulation of CD107a expression in NKG2A^+^KIR^-^ subset was detected only after stimulation with 221-B*51:01-shERAP1 compared with 221-B*51:01-shCTRL (**Figures 5A, B**). The mean frequency of CD107a cells was 25.2 ± 10.2 *vs* 22.9 ± 10.7 for 221-A2-shERAP1 *vs* 221-A2-shCTRL, 55.1 ± 17.6 *vs* 40.8 ± 22.3 for 221-B*51:01-shERAP1 vs 221-B*51:01-shCTRL; 13.9 ± 14.2 *vs* 18.8 ± 15.3 for 221-Cw3shERAP1 *vs* 221-Cw3shCTRL, 20.8 ± 12.2 *vs* 25.1 ± 14.2 for 221-Cw4-shERAP1 *vs* 221-Cw4-shCTRL, and 53.2 ± 12.9 *vs* 50.5 ± 12.3 for 221-Cw7-shERAP1 *vs* 221-Cw7shCTRL (**Figure 5B**). Thus, ERAP1 inhibition results in increased NK cell-mediated killing of 221-B*51:01 cells due to impaired engagement of both KIR3DL1 and NKG2A.

### Pharmacological Inhibition of ERAP1 Mirrors Its Genetic Depletion by Enhancing NK Cell-Mediated Killing of 221-B*51:01 Cells

**Figure 6:**
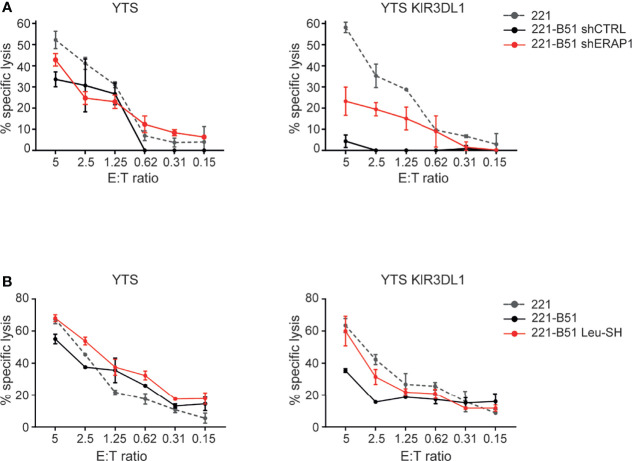
Genetical and pharmacological inhibition of ERAP1 makes 221-B*51:01 cells more susceptible to lysis by NK cells expressing KIR3DL1*001. **(A, B)** 221 transfectant cells genetically **(A)** or pharmacologically **(B)** inhibited for ERAP1 were incubated with YTS or YTS-KIR3DL1*001 NK cells. A representative experiment of three independently performed is shown. Data represented mean ± SD of triplicate.

The effect of ERAP1 inhibition on the KIR3DL1-HLA-B*51:01 interaction was further assessed by comparing the ability of the NK cell line YTS, both KIR and NKG2A negative, and YTS overexpressing KIR3DL1 (**Figure S2**) to 221 and 221-B*51:01 in a cytotoxic assay. As expected, 221 cells were efficiently lysed by both YTS and YTS-KIR3DL1, whereas 221-B*51:01-shCTRL were lysed by parental YTS, but protected from lysis by YTS-KIR3DL1. Genetic depletion of ERAP1 abrogated KIR3DL1-mediated protection by YTS-KIR3DL1 (**Figure 6A**). Similar results were obtained by pharmacological inhibition of ERAP1 (**Figure 6B**), demonstrating that ERAP1 is critical for recognition of HLA-B*51:01 by KIR3DL1.

## Discussion

ERAP1 plays a crucial role in immune surveillance by trimming peptides with the correct length and composition to be presented by MHC class I molecules. Our previous studies (27, 28) revealed that ERAP1 controls the interaction of inhibitory KIRs with their specific ligands in some tumor cells and lymphoblastoid cell lines. Using the 221 cell model over-expressing the ligands of KIR2DL1, KIR2DL3 and KIR3DL1, we show that genetical and pharmacological inhibition of ERAP1 specifically impairs the interaction of KIR3DL1 with its ligand HLA-B51 making 221-B*51:01 cells more susceptible to being killed by the NK cell subset expressing KIR3DL1. In addition, inhibition of ERAP1 also made 221-B*51:01 cells more susceptible to lysis by NKG2A-expressing NK cells.

The increased activation of KIR3DL1^+^ NK cell subset against 221-B*51:01-shERAP1 cells could depend to an altered peptide repertoire presented on the cell surface. Accordingly, analysis of the MHC class I immunopeptidome showed that loss of ERAP1 completely changes the repertoire of peptides, some being dramatically upregulated and others completely lost (34). Similar results were obtained for several HLA class I alleles, including HLA-B51 (33). In particular, Lopez De Castro’s group demonstrated that the B51 immunopeptidome is profoundly affected by ERAP1 (33, 35). The immunopeptidome resulting in the absence of ERAP1 showed lower binding affinity to HLA-B51 molecules. These data support our findings indicating that the immunopeptidome derived from inhibition of ERAP1 impairs the interaction of HLA-B51 molecules with the KIR3DL1 receptor.

The increased activation of the NKG2A^+^ NK cell subset against 221-B*51:01-shERAP1 cells following ERAP1 inhibition might instead depend on the contribution of ERAP1 in cleaving the signal sequence of HLA class I molecules. The signal sequence of the various HLA class I molecules differs in some amino acid positions (**Table S3**). The nature of these amino acids might influence the contribution of ERAP1 in the cleavage of peptide ligands of HLA-E. Indeed, ERAP1 is known to select peptides based on their amino acid composition: residues at the carboxy-terminal determine selectivity for the enzyme, whereas those at the aminoterminal determine trimming specificity (36, 37).

A possible hypothesis to explain how receptor engagement is affected by peptide-selective conformational changes is that they are transmitted from the peptide-filled groove to the HLA molecule/KIR interface through a “domino” effect involving successive positional adjustments (38). Based on these results, therapeutic approaches aimed at modulating ERAP1 functions by resulting in a change in the repertoire of peptides presented by MHC class I, could be more than promising for activating NK cells.

NK cell-based immunotherapy represents an interesting approach for adjuvant treatment of many types of cancers. One of the most promising settings to test the adoptive infusion of allogeneic NK cells is HSCT (29). The use of donors with alloreactive NK cells displaying anti-leukemia activity is associated with a lower risk of recurrence in leukemia patients undergoing haploidentical HSCT, without increasing the risk of graft-versus-host disease (GVHD) (39, 40). The success of this approach is explained by the transplantation of donor-derived alloreactive NK cells, which persist for years in patients after haploidentical HSCT, contributing to the eradication of leukemic cells (41). However, since not all the patients have the possibility to receive a transplant from an alloreactive donor, there is an urgent need to identify new therapeutic strategies able to induce or enhance NK cell alloreactivity.

A large variety of new drugs able to enhance NK cell response are being tested in clinical trials, but a toxic effect has been reported for some of them (42, 43). Development of small molecules targeting ERAP1 might provide an innovative tool to improve outcome of NK cell-based antitumor therapy protocols. Leu-SH is a potent inhibitor of ERAP1 activity and it has been successfully used to reproduce the effects of genetic ERAP1 suppression (28) albeit it is not ERAP1 specific. A novel class of more selective inhibitors has been recently described (44–47). These new compounds are effective in targeting *in vitro* ERAP1 inside the ER at the nmol/L level, and modulate cytotoxic T cell responses, suggesting their potential use for pharmacologic manipulation of NK cell and T cell antitumor activity.

Taken together, our results suggest that inhibition of ERAP1 function may have important therapeutic applications in NK cell-based cancer immunotherapy. Individuals carrying HLA-B*51:01-like antigens and active ERAP1 haplotypes may be eligible for therapies based on ERAP1 inhibition.

## Data Availability Statement

The original contributions presented in the study are included in the article/**Supplementary Material**. Further inquiries can be directed to the corresponding author.

## Author Contributions

Conceptualization, SD’A, VD’A, MC, and DF. Methodology, SD’A, VD’A, MC, MF, LC, DP, and DF. Validation, PT, GG, PR, VL, OM, and MA. Formal analysis, investigation, resources, and data curation, SD’A, VD’A, MC, PT, GG, PR, VL, OM, LC, and MA. Writing—original draft preparation, SD’A and DF. Supervision, DF. Funding acquisition, DF. All authors contributed to the article and approved the submitted version.

## Funding

This work was supported by grants awarded by H2020-MSCA-ITN-2020 Capstone-954992, Ministero della Salute Ricerca Finalizzata PE-2011-02351866, Associazione Italiana Ricerca sul Cancro (AIRC) IG18495 and IG24345, and Ricerca Corrente (DF). This research was also supported by two fellowships from the Fondazione Veronesi (VL and OM).

## Conflict of Interest

The authors declare that the research was conducted in the absence of any commercial or financial relationships that could be construed as a potential conflict of interest.

## Publisher’s Note

All claims expressed in this article are solely those of the authors and do not necessarily represent those of their affiliated organizations, or those of the publisher, the editors and the reviewers. Any product that may be evaluated in this article, or claim that may be made by its manufacturer, is not guaranteed or endorsed by the publisher.
