# Supplementary material for: ERAP1 Controls the Interaction of the Inhibitory Receptor KIR3DL1 With HLA-B51:01 by Affecting Natural Killer Cell Function
Source: Front Immunol. 2021 Nov 30;12:778103. doi: 10.3389/fimmu.2021.778103 (PMC8669763; doi:10.3389/fimmu.2021.778103)
Supplement: Supplementary file 1 [file DataSheet_1.pdf]

Table S1. ERAP1 allotypes in 221 cell line

Exons	SNP	AA substitution*	AA residue
**2**	rs3734016	E56K	E
**2**	rs26653	R127P	P
**5**	rs26618	I276M	M, I
**6**	rs27895	G346D	G
**6**	rs2287987	M349V	M
**11**	rs30187	K528R	K, R
**12**	rs10050860	D575N	D
**15**	rs17482078	R725Q	R
**15**	rs27044	Q730E	E, Q

*Amino acid residue numbering and consensus sequence are from human ERAP1 isoform 2 (UniProt Q9NZ08-2).

Table S2. List of primers for ERAP1 genotyping

Sequencing primers	SNP
5’-ATGGTGTTTCTGCCCCTCAAATGGT-3’	rs26653, rs3734016
5’-GAAGATGAGCACCTATCTG-3’	rs26618
5’-GCTCTGTTGTTTGATGCAG-3’	rs27895, rs2287987, rs30187
5’-GTACACATGAAGCAAGAGCA-3’	rs10050860, rs17482078, rs27044

Table S3. List of HLA class I derived signal sequences

HLA class I allele	Signal sequence peptides
HLA-B51	MRVTAPRTVLLLLWGAVALTETWA
HLA-A2	MAVMAPRTLLLLLSGALALTQTWA
HLA-Cw3	MRVMAPRTLILLLSGALALTETWA
HLA-Cw4	MRVMAPRALLLLLSGGLALTETWA
HLA-Cw7	MRVMAPRALLLLLSAALALTETWA

Differences between the signal sequence of HLA-B51 and those of the other class I alleles are highlighted. In grey: putative HLA-E peptide ligand.
