# Supplementary figures and images for: ERAP1 Controls the Interaction of the Inhibitory Receptor KIR3DL1 With HLA-B51:01 by Affecting Natural Killer Cell Function
Source: Front Immunol. 2021 Nov 30;12:778103. doi: 10.3389/fimmu.2021.778103 (PMC8669763; doi:10.3389/fimmu.2021.778103)

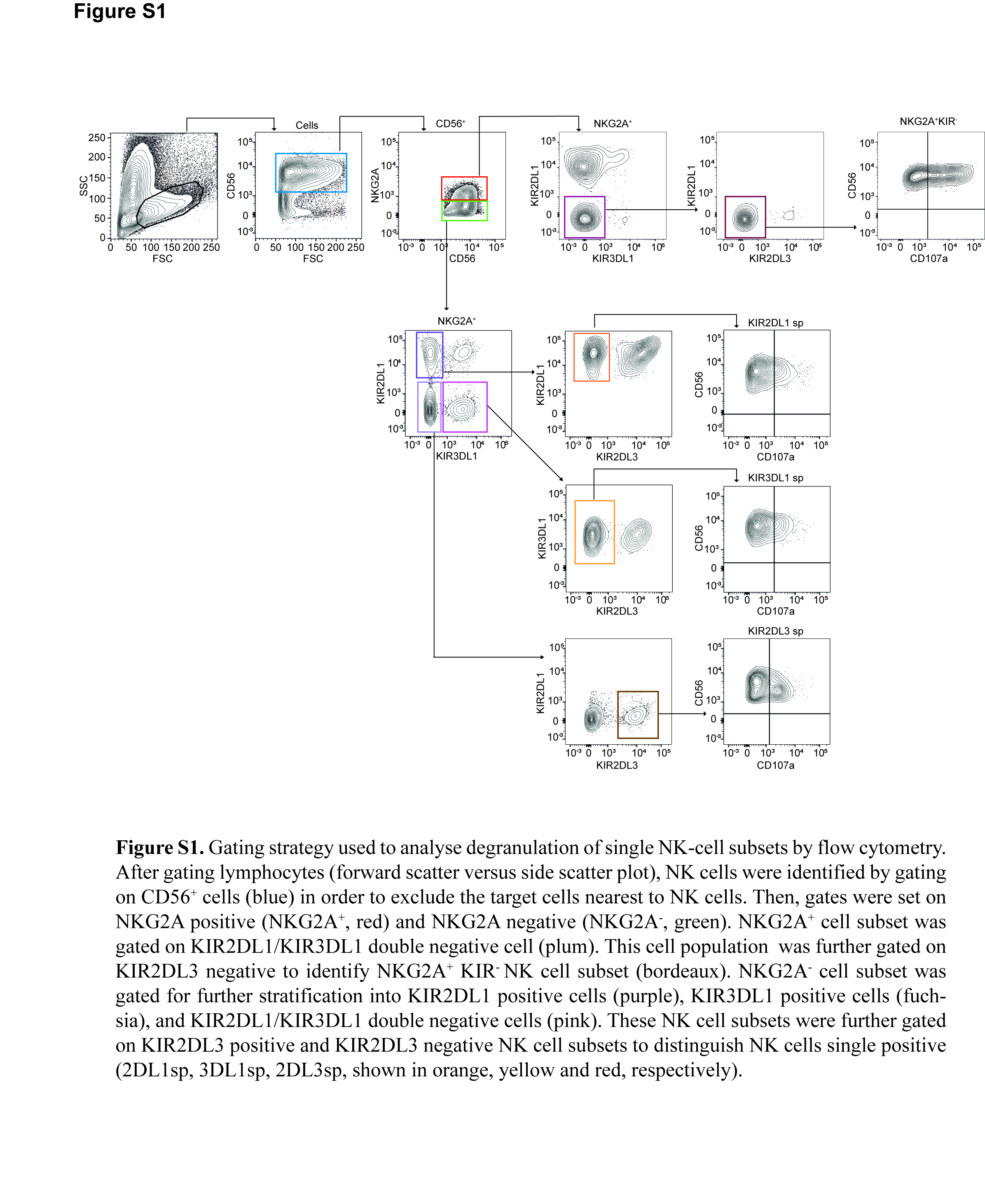

Supplement: Supplementary file 2 [file Image_1.tif]

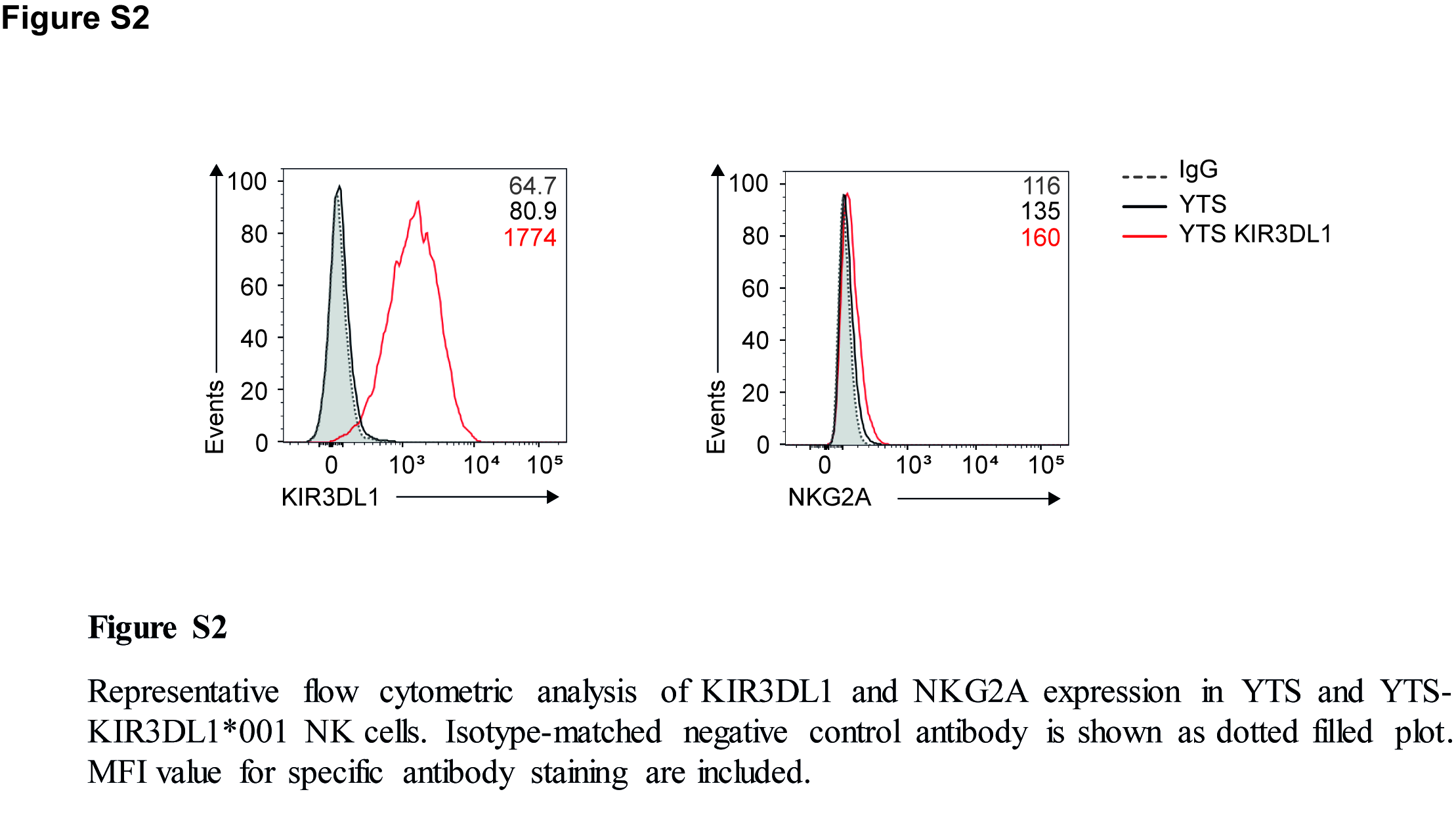

Supplement: Supplementary file 3 [file Image_2.tif]
